# Essential Oils and Their Constituents: An Alternative Source for Novel Antidepressants

**DOI:** 10.3390/molecules22081290

**Published:** 2017-08-03

**Authors:** Damião P. de Sousa, Rayanne H. N. Silva, Epifanio F. da Silva, Elaine C. Gavioli

**Affiliations:** 1 Departamento de Ciências Farmacêuticas, Universidade Federal da Paraíba, João Pessoa, PB 58051-970, Brazil; damiao_desousa@yahoo.com.br (D.P.d.S.); rayanne_nasci@hotmail.com (R.H.N.S.); 2 Departamento de Biofísica e Farmacologia, Universidade Federal do Rio Grande do Norte, Natal, RN 59078-970, Brazil; epifaniofsilva@hotmail.com

**Keywords:** oil, terpene, natural products, major depression, antidepressant, animal models

## Abstract

Depression is a disease that has affected a high proportion of the world’s population and people of different ages, incapacitating them from good performance at work and in social relationships, and causing emotional disorders to millions of families. Therefore, the search for new therapeutic agents is considered a priority for the discovery of more effective forms of treatment. In this review, studies of essential oils and their constituents in experimental models related to depression are discussed. The mechanisms of action of the oils and the presence of psychoactive constituents in their chemical compositions are discussed. The data in the review show the therapeutic potential of essential oils and their chemical constituents for use in depressive disorders. Advanced studies using humans are needed to confirm the antidepressant properties described in animals.

## 1. Introduction

Depression is one of the most prevalent and costly psychiatric disorders; it leads to substantial cognitive and affective disturbances, and negatively impacts the overall quality of life. Major depression is manifested through psychological, behavioral and physiological symptoms, comprised of depressed mood, markedly diminished pleasure in most activities, loss of energy, poor concentration, alterations in appetite and sleeping patterns, feelings of worthlessness, excessive guilt, and thoughts of death or suicide [1]. A systematic review has predicted an average prevalence for major depression at a global level of 4.7% [2]. This means that one out of every 20 people in the world is affected by depression. The prevalence estimated for depression in women was 5.9% and 3.8% for men [2]. Women are thus almost twice as likely to suffer from major depression as men.

## 2. Pharmacological Management of Major Depression

Conventional antidepressant drugs ultimately act by increasing monoamine levels at the synaptic cleft by either: (i) blocking presynaptic monoamine transporter proteins, which remove released transmitters from the extracellular space; (ii) inhibiting the enzyme monoamine oxidase, which degrades monoamine neurotransmitters; or (iii) interacting with pre- or postsynaptic receptors that regulate monoamine transmitter release and/or neuronal firing rate [3]. It has been proposed that as antidepressant drugs increase extracellular monoamine concentrations, depression might be produced by deficiencies in noradrenaline, 5-HT and dopamine at their receptor sites in the brain. This proposal is known as the monoamine depression hypothesis [4]. Although the effects of antidepressants on monoamines can be seen soon after administration, it generally takes a few weeks of continued treatment for therapeutic responses to appear. Due to the therapeutic delay of antidepressants, problems involving the neural network’s processing of information, rather than chemical disequilibrium, might well underlie depression [4]. In fact, conventional antidepressants mediate their effects by increasing Brain-Derived Neurotropic Factor (BDNF) in the forebrain regions, particularly in the hippocampus, making BDNF an essential determinant of antidepressant efficacy. BDNF acts in the brain inducing neuroplasticity, which results in depressive symptom improvements [5], and it has already been shown that hippocampal neurogenesis is a requirement for the therapeutic effects of antidepressants [6].

Depression pharmacotherapy is costly, though widely prescribed by physicians. However, less than half of the patients treated obtain complete remission through therapy with single antidepressant drugs. Some patients exhibit partial or no remission and some patients display treatment intolerance responses. This emphasizes the need to identify novel classes of antidepressants [7]. The most frequent side effects of these drugs are due to rapid monoamine concentration increases at the receptor sites. These effects could be summarized as increased anxiety, gastrointestinal and sexual problems and decreased alertness. The challenge for such new antidepressants is to achieve fast antidepressant response, broader efficacy, and fewer adverse effects [7].

Being a rich source for bioactive molecules, medicinal plants provide hope for development of novel antidepressant drugs [8,9,10]. An alternative approach might come from aromatic plants. Although certain essential oils found in plants have been used as traditional medicines, little scientific evidence supports their use. Essential oils are complex mixtures of volatile compounds produced by aromatic plants [11]. Recent clinical studies show that essential oils, inhaled or orally administered, enter the blood stream and exert psychological effects, thus complementing pharmacodynamic mediation. For instance, inhalation, or oral administration of essential oils improves the quality of sleep [12,13], attenuates symptoms of dementia [14,15], negative affect [16], anxiety [11,17], nicotine craving [18], post-traumatic stress disorder [19] and Alzheimer’s disease [20]. Preclinical pharmacological studies of essential oils and/or their isolated chemical constituents are becoming more common [21,22,23,24]. In fact, several studies have shown that the aromatherapy could to be used as a complementary and alternative therapy for patients with depression and secondary depressive symptoms [25], including anxiety disorders [26]. In a review published on plants used in aromatherapy for anxiety treatment, the contributions of the chemical constituents of their essential oils in this therapeutic effect are discussed [27], while a recent systematic review discusses the anxiolytic action of essential oils and their constituents [11]. The objective for this review is to discuss certain essential oils and their isolated constituents being tested in humans and rodents for treatment of major depression. These essential oils and their constituents have been pre-clinically tested for their antidepressant activity and are, respectively, illustrated in Table 1 and Table 2. In addition, the proposed mechanisms by which essential oils and their isolated compounds produce antidepressant actions are also discussed. Considering all of the information herewith presented, essential oils might well be an alternative source of therapy for the relief of major depression symptoms.

## 3. Methodology

The present study was carried out based on the literature review of plants and their essential oils with antidepressant activity. Chemical structure and name of bioactive compounds, as well as references are also provided. All species mentioned in the text were validated taxonomically on Database (www.theplantlist.orgW3Tropicos).

The plant species presented here were selected based on the effects shown by their essential oils in specific animal models used for evaluation of antidepressant activity and/or by complementary studies, aimed at elucidating the mechanism(s) of action of the oils or individual components. To select the essential oil constituents, terms related to the theme, such as “essential oils”, “monoterpenes” and “phenylpropanoids”, were used, as well as names of representative compounds of these chemical groups refining with “antidepressant” or “depression”. A search was performed in the scientific literature database PubMed from 1995 to December 2015. The essential oils or the main constituents were deemed to display antidepressant activity when they had shown effects in one or more different depressant model. The scientific publications were selected from studies published in English language.

## 4. Clinical Effects of Essential Oils on Mood Depression

Certain clinical studies have been aimed at investigating the effects of essential oils in humans on mood and major depression. The most frequently studied essential oil for mood states is lavender, possibly due to its previously well-recognized anxiolytic effects [17]. Lavender oil capsules produced from *Lavandula angustifolia* Mill. (Lamiaceae) flowers were tested as an adjuvant therapy for major depression in patients under conventional pharmacological treatment [28]. In this pilot study, eight patients diagnosed with major depression and symptoms of anxiety, insomnia, and psychomotor agitation were treated with lavender oil for three weeks. The results demonstrated that lavender oil reduced certain anxiety related symptoms, psychomotor agitation, and sleep disturbances in the depressed patients, thus indicating significant improvements as compared to classical antidepressant medication alone [28].

The acute inhalation effects of lavender essential oil were investigated on moods in adult healthy men [29]. Electroencephalogragy (EEG) activity, alertness, and mood were assessed in 40 healthy adult men given 3 min (daily) of lavender oil inhalation. The subjects showed increased EEG beta power, reported feeling more relaxed, and with less depressive moods (as scaled by Profile of Mood States (POMS)); they also performed math computations faster and more accurately [29]. These findings suggest that lavender oil can improve moods even in healthy individuals.

In another clinical pilot trial, the effects of an essential oil blend of *Lavandula angustifolia* Mill. and *Rose otto* (syn. *Rosa* × *damascena* Mill.), Rosaceae, in 28 postpartum women diagnosed with mild to moderate depression or anxiety [30]. The essential oil was administered by inhalation or using the dermal route (in a white lotion). Treatment consisted of 15 min sessions, twice a week for four consecutive weeks. The essential oils significantly relieved both depression symptoms (as scored by Edinburgh Postnatal Depression Scale (EPDS)), and anxiety (as scored by Generalized Anxiety Disorder Scale (GAD-7)). There were no adverse effects reported [30]. The study supported the beneficial effects of essential oils, in this case a combination of rose and lavender oils, for relief of depression and anxiety in postpartum women. Ultimately, this clinical study proposed an interesting approach combining distinct essential oils to better treat psychiatric disorders and/or comorbid diseases.

*Salvia sclarea* L. (Lamiaceae) essential oil was tested in a pilot trial for modulation of depression signs in 22 women [31]. Normal and depressive tendencies and serum parameters in menopausal women acutely inhaling clary sage oil were assessed before and after exposition. Given the comparison between pre-inhalation and post-inhalation of clary sage oil, 5-HT plasma concentrations increased significantly, and plasma cortisol levels decreased significantly for both normal and depressive menopausal women [31]. It should be mentioned that this pioneering clinical trial contributed by measuring physiological changes alongside of behavioral alterations in women with depressive symptoms after acute clary sage oil inhalation.

In a controlled, and randomized clinical pilot trial, the effect of continuous inhalation, in adult men with depression and under conventional pharmacological treatment, of citrus fragrance, (whose main component was lemon oil), was compared with that of no fragrance (n = 12 and 8, respectively) [32]. Four to eleven weeks of citrus fragrance inhalation significantly improved mood states, as scored by the Symptom Distress Scale (SDS) and the Hamilton Rating Scale for Depression (HRSD). The results indicated that therapeutic dosages necessary for treatment of depression can be markedly reduced. In fact, treatments with citrus oil normalized neuro-endocrine hormone levels and immune function [32]. This distinctly long-term clinical trial of citrus fragrance brings considerable insight to the beneficial effects of essential oils as therapy adjuvants for the treatment of major depression.

## 5. Antidepressant-Like Effects of Essential Oils: Evidence from Animal Studies

As summarized in Table 1, the following essential oils of plants displayed some antidepressant-like effects when tested in rodents: essential oils of *Acorus tatarinowii* Schott (Acoraceae), *Asarum heterotropoides* F. Schmidt (Aristolochiaceae), *Citrus limon* (L.) Osbeck (Rutaceae), *Eugenia uniflora* L. (Myrtaceae), *Lavandula angustifolia* Mill, *Litsea glaucescens* Kunth (Lauraceae), *Mentha* × *piperita* L. (Lamiaceae), *Perilla frutescens* (L.) Britton (Lamiaceae), *Rosmarinus officinalis* L. (Lamiaceae), *Salvia sclarea* L., *Schinus terebinthifolius* Raddi (Anacardiaceae), *Syzygium aromaticum* (L.) Merr. & L.M. Perry (Myrtaceae), *Toona ciliata* Roem var. Yunnanensis (C. DC.) C.Y. Wu (Meliaceae), *Valeriana wallichii* DC. (Caprifoliaceae), and *Zingiber officinale* Roscoe (Zingiberaceae). The most promising aromatic plants with significant evidence of antidepressant-like effects and the putative mechanisms by which they act are detailed below. Indeed, the main constituents of some of these essential oils have been already isolated, identified, and even tested for antidepressant effects in rodents.

### 5.1. Asarum heterotropoides *F. Schmidt (Aristolochiaceae)*

Recently, a study showed for the first time the antidepressant-like effects of *Asarum heterotropoides* F. Schmidt essential oil (from the roots) in mice [34]. The chemical composition of this essential oil was analyzed; 78 peaks were detected by gas chromatography. The main compounds are methyl eugenol (22%), pentadecane (6%), and 2,3,5-trimethoxytoluene (5%). Antidepressant-like effects were observed after acute inhalation in behavioral despair assays (e.g., forced swimming and tail suspension tests) in mouse. Considering the prevalence of methyl eugenol in the *Asarum heterotropoides* F. Schmidt essential oil, and the antidepressant-like actions previously reported about this compound in rats [50], it might be suggested the methyl eugenol as the main mediator of the antidepressant effects induced by *Asarum heterotropoides* F. Schmidt. Immunohistochemistry was performed to investigate the mechanisms of action. An increase in the CRF- and tyrosine hidroxylase-positive cells in the paraventricular nucleus and locus coeruleus, respectively, and a significant decrease of 5-HT-positive cells was observed in the mouse dorsal raphe after forced swimming exposure. The inhalation of *Asarum heterotropoides* F. Schmidt essential oil restored to normal levels the immunoreactivity to 5-HT, CRF and tyrosine hidroxylase. Considering that stress-induced depression-like behaviors are closely linked to increased activity of the endogenous peptidergic system of CRF and reduced availability of monoamines [51], the mechanistic findings herein reported could be on the basis of the antidepressant-like effects of *Asarum heterotropoides* F. Schmidt oil.

### 5.2. Citrus limon *L. Osbeck*

Acute inhaled lemon oil reduced immobility time in the FST in rats and mice [35,36]. However, in the same studies, inhalation of this oil reduced locomotion and exploration in the open field, which would be suggestive of a sedative effect [35,37]. Later, Lopes et al. [37] evaluated prolonged oral effects of *Citrus limon* L. Osbeck oil (from the leaves) in mice in the FST. Antidepressant, anxiolytic and hypolocomotor effects in animal were reported in a dose-dependent manner. A mixture of monoterpenes was detected in the *Citrus limon* L. Osbeck essential oil, among which limonene (53%), geranyl acetate (10%) and *trans*-limonene-oxide (7%) were the main compounds [37]. Contrasting findings are available in literature regarding the effects of limonene on mood states in rodents. After acute administration, the monoterpene was inactive in the mouse FST [40]. However, under prolonged treatment (15 days), limonene reversed increased immobility time in the FST induced by neuropathic pain in rats [46]. The putative mechanism by which lemon oil produces antidepressant-like effects seems to be mediated by 5-HT and dopamine neurotransmission. The pretreatment with buspirone (5-HT_1A_ partial agonist), DOI (5-HT_2A_ receptor agonist), miaserin (5-HT_2A/C_ receptor agonist), apomorphin (nonselective dopamine receptor agonist) and haloperidol (nonselective dopamine receptor antagonist), blocked the antidepressant effects of lemon oil [35]. Moreover, the acute inhalation of this oil significantly increased dopamine contents in the hippocampus and 5-HT in the prefrontal cortex and hippocampus [35]. As commented before, dopamine and 5-HT are intrinsically involved in the modulation of mood states, and hippocampus and prefrontal cortex are the main stages of this action [4]. Thus, the antidepressant-like effects of *Citrus limon* L. Osbeck oil might be mediated by limonene. Indeed, modulation of 5-HT and dopamine neurotransmission in brain areas highly involved with mood states could be on the basis of the antidepressant effects of lemon oil.

### 5.3. Eugenia uniflora *L.*

The potential antidepressant-like effects of *Eugenia uniflora* L. essential oil showed in a dose-dependent manner after acute administration in the TST in mice [38]. The chemical composition of *Eugenia uniflora* L. oil was analyzed by gas chromatography/mass spectroscopy; and it contains mainly sesquiterpenes: germacrene B (22%), selina-1,3,7-trien-8-one-oxide (19%), β-caryophyllene (13%), germacrene A (11%), germacrene D (11%), selina-1,3,7-trien-8-one (9%) and curzerene (4%). Only β-caryophyllene has been tested for the effects on depression states. The acute administration of β-caryophyllene induced robust antidepressant-like effects, as replicated in distinct animal models in mice: FST, TST, and novelty-suppressed feeding behavior [52]. Indeed, the antidepressant effects of the isolated constituent β-caryophyllene were prevented by the pretreatment with a CB_2_ receptor antagonist, AM630 [52]. Interestingly, β-caryophyllene acts as a CB2 receptor agonist [53]. The CB_2_ receptor is expressed also in the brain, and is involved in the modulation of anxiety and depressive states [54]. Victoria et al. [38] showed the involvement of monoamines neurotransmission mediating the *Eugenia uniflora* L. oil-induced antidepressant actions. The blockade of 5-HT_2A/C_, α_1_- and α_2_-receptors prevented the antidepressant effects of this essential oil in the mouse TST. Additional studies aimed to investigate the effects of chronic administration of *Eugenia uniflora* L. oil in animal models of depression and putative mechanisms of action are worth carrying out.

### 5.4. Perilla frutescens *L. Britton*

Two distinct research groups from China have described the antidepressant effects of *Perilla frutescens* L. Britton essential oil in mice. Using the chronic unpredictable mild stress (CUMS), a well validated animal model of depression, these studies showed the effects of the essential oil in reversing behavioral, neurochemical, and immunological alterations induced by stress [42,43]. The essential oil restored sucrose-preference in stressed mice, a behavior intrinsically related to anedonia, a core symptom of depression. In addition, the treatment with this oil also restored the increased immobility time in the FST and TST in CUMS mice, thus supporting a robust antidepressant-like action [42,43]. Changes in 5-HT and BDNF levels might be based on the antidepressant actions.

The administration of the essential oil effectively reversed the reduced hippocampal concentrations of 5-HT, its metabolite, 5-HIAA, and BDNF protein and mRNA [42,43]. A growing body of evidence supports the release of pro-inflammatory cytokines, mainly IL-1β, IL-6, and TNF-α, in major depression [55]. The chronic administration of *Perilla frutescens* L. Britton oil dose-dependently decreased the serum IL-6, IL-1β, and TNF-*α* levels in CUMS-mice. The main constituents of this essential oil extracted by supercritical fluid are l-perillaldehyde, limonene, beta-caryophyllene, selinene, santalene and bergamotene [56]. l-perillaldehyde-induced antidepressant-like effects have already been reported in mice [42,57]. Repeated administration (oral and inhalated) of this compound reversed depressant-like behaviors induced by CUMS and lipopolyssacaride (LPS) [42,57]. Concerning the mechanism of action of l-perillaldehyde on depressive states, restored concentrations of 5-HT and noradrenaline in the prefrontal cortex were observed in LPS-treated mice, and attenuated LPS-induced increases of TNF-α and IL-6 levels [42]. The *Perilla frutescens* L. Britton oil has other compounds with antidepressant-like actions, such as limonene and beta-caryophyllene [46,52]. Taken together, the robust antidepressant effects of *Perilla frutescens* L. Britton oil suggest that more than one active compound, with distinct mechanisms of action, could be mediating this effect.

### 5.5. Salvia sclarea *L.*

The antidepressant effects of essential oil of *Salvia sclarea* L. were assessed in the FST in rats. The acute exposition to this oil, via intraperitoneal and inhalation, reduced immobility time similar to conventional antidepressant drugs [39]. The antidepressant effects of this essential oil seem to be mainly mediated by the activation of dopamine and 5-HT neurotransmission [39]. In fact, the pretreatment with haloperidol (Dopamine receptor antagonist), SCH-23390 (D_1_ receptor antagonist), but also buspirone (5-HT_1A_ partial agonist) blocked the antidepressant effect of this essential oil [39]. The principal constituents of *Salvia sclarea* L. oil include linalyl acetate (64%), linalool (21%), and geraniol (2.6%) [32]. Linalool and geraniol have showed consistent antidepressant actions in rodents after acute administrations [40,58,59,60]. This effect of linalool in rodents were prevented with WAY100,635 (5-HT_1A_ receptor antagonist) and yohimbine (α_2_-receptor antagonist), thus reinforcing the role mediated by monoaminergic neurotransmission in the antidepressant effects of linalool [58]. Ultimately, the antidepressant of the *Salvia sclarea* L. essential oil seems to be due to the synergic effects of bioactive isolated compounds.

### 5.6. Syzygium aromaticum *(L.) Merr. & L.M. Perry*

Recently, a well-designed study showed the antidepressant effects of *S. aromaticum* essential oil in rodents. After acute administration, this essential oil reduced immobility time in mice in the FST and TST. Using the CUMS, chronic administration of *S. aromaticum* (L.) Merr. & L.M. Perry oil restored sucrose preference and reversed the increased latency to feed in an unfamiliar environment in CUMS rats [47]. The antidepressant doses (50–200 mg/kg) were, at least, 22-fold higher than the lethal dose 53 (LD_50_ = 4.5 g/kg). The chronic administration of this essential oil restored hippocampal BDNF, p-ERK and p-CREB protein expression in CUMS rats [47]. The major compounds identified in *S. aromaticum* (L.) Merr. & L.M. Perry essential oil were eugenol (71%), β-caryophyllene (10%), eugenyl acetate (16%) [47]. Literature findings support antidepressant-like actions for eugenol and β-caryophyllene [52,61,62], which could be synergically mediating the antidepressant effects of the *S. aromaticum* (L.) Merr. & L.M. Perry oil. Repeated administration of eugenol reduced immobility in the TST and FST [61,62]. The antidepressant effects of eugenol were attributed to the inhibition of human MAO_A_ [60], and the increase in hippocampal BDNF [61]. These findings suggest that eugenol, but also β-caryophyllene could be mediating the antidepressant-like actions of *S. aromaticum* (L.) Merr. & L.M. Perry essential oil by the increase in monoamine neurotransmission and neuroplastic actions.

### 5.7. Toona ciliata *var.* yunnanensis *(C. DC.) C.Y. Wu*

The acute administration of *T. ciliata* var. Yunnanensis (C. DC.) C.Y. Wu oil evoked antidepressant-like actions in a dose-dependent manner in mice, in the FST and TST [48]. In addition, the treatment with this essential oil increased hippocampal monoamines (5-HT, noradrenaline and dopamine) and BDNF contents in CUMS rats [48]. The major compounds identified in *T. ciliata* var. Yunnanensis (C. DC.) C.Y. Wu oil by gas chromatography/mass spectroscopy were β-elemene (25%), β-cubebene (14%), γ-elemene (8%), and estragole (6%) [48]. None of these components have been tested yet for the effects on depressive states. Further studies aimed to evaluate the effects of the isolated oil compounds and the *T. ciliata* var. Yunnanensis (C. DC.) C.Y. Wu oil during chronic administration in animal models of depression are warranted.

### 5.8. Valeriana wallichii *DC.*

The effects of *Valeriana wallichii* DC. (patchouli alcohol chemotype) were tested in the mouse FST after acute and 14-days administration [49]. The acute treatment with this essential oil reduced, in a dose-dependent manner, the immobility time of mice in the FST; the antidepressant doses in this assay were 20 mg/kg and 40 mg/kg [49]. However, after repeated administration, the *V. wallichii* DC. oil reduced immobility time only at 20 mg/kg [49]. Chronic administration increased norepinephrine and 5-HT levels in the mouse brain [61]. More evidence suggests the participation of nitric oxide signaling pathway in the acute antidepressant-like effect of *V. wallichii* DC. essential oil. The pretreatment with l-arginine (NO precursor) and sildenafil (phosphodiesterase 5 inhibitor) prevented the antidepressant effect, while it was potentiated with L-NAME (NOS inhibitor), and methylene blue (inhibitor of soluble guanylate cyclase) [49]. These findings are in accordance with previous studies that showed reduction of NO levels within the brain inducing antidepressant-like effects [63,64,65]. Classical antidepressant drugs induce behavioral effects in the FST via blockade of nitregic system pathway [66,67]. The *V. wallichii* DC. oil constituents were identified by gas chromatography-mass spectroscopy. The oil contains patchouli alcohol (40%) as the major constituent followed by the presence of δ-guaiene (10%), seychellene (8%), 8-acetoxyl patchouli alcohol (4%) and virdiflorol (5%). These isolated compounds have not still tested on experimental depression. Further studies, aimed at investigating the effects of these isolated compounds as well as the effects of *V. wallichii* DC. essential oil on depression in rodents, are needed [49].

## 6. Constituents from Essential Oils with Antidepressant-Like Activity

The effects of isolated compounds from essential oils in the rodent behavior are summarized in Table 2.

### 6.1. Isolated Constituents with Proposed Mechanisms of Antidepressant Action

The most studied isolated compounds are eugenol [61,62] and linalool [40,58,59]. Studies suggest robust antidepressant-like actions as demonstrated in distinct behavioral tests (FST and TST) performed in different labs around the world. The main target of the antidepressant action of eugenol is the MAO enzyme [62]. This compound preferentially inhibits the MAO_A_ activity, and after chronic administrations increases the neurotrophic factor, BDNF, a mechanism of action shared with conventional antidepressants [4]. Concerning linalool, main constituent of the extracted lavender and clary sage oil [31,76], acute studies suggestive of antidepressant actions support the activation of monoamine 5-HT_1A_ and α_2_-receptors [58].

Other isolated constituents from essential oils that induce antidepressant-like actions possibly mediated by monoamines are l-menthone [72], perillaldehyde [57], thymol [73] and thymoquinone [74]. These compounds increase monoamines in the brain, a mechanism of action similar to classical antidepressants. Studies showed that carvacrol and β-pinene induce antidepressant-like actions in behavioral despair assays, e.g., FST and TST [58,68]. The acute treatment with these isolated constituents decrease the immobility time, an effect reversed by the pretreatment with SCH23390, a dopamine D_1_ antagonist [58,68]. The antidepressant effects of β-pinene were also blocked by 5-HT_1A_ antagonist, β-antagonist and DSP-4, a noradrenergic neurotoxin [58]. By contrast, the pretreatment with prazosin, a α_1_-receptor antagonist, yohimbine, a α_2_-receptor antagonist, and PCPA, a 5-HT synthesis inhibitor, did not affect the antidepressant effects of carvacrol [68]. These experimental data suggest that β-pinene seems to evoke antidepressant actions dependent on dopaminergic, serotoninergic and noradrenergic systems, while carvacrol displays antidepressant activity mainly mediated by dopaminergic system.

Some other promising isolated compounds from essential oils, such as l-menthone [72], thymol [73] and geraniol [47] induce antidepressant-like effects in rodents and the mechanisms of these actions were partially described. For these compounds, potential anti-inflammatory effects may be involved and/or mediating the antidepressant actions. A growing number of preclinical and clinical studies have demonstrated an association between concentrations of pro-inflammatory cytokines—mainly interleukin (IL)-1β, IL-6, and tumor necrosis factor-α and depressive symptoms. In addition, mounting evidence has shown a concomitant reduction in both depressive symptoms and pro-inflammatory cytokine concentrations following treatment with anti-inflammatory drugs [77]. In this view, l-menthone, thymol and geraniol inhibited IL-1β, IL-6 and TNFα cytokines and other pro-inflammatory intracellular signaling, such as NF-κB, NLRP3 and caspase 1, usually increased by stress. Interestingly, the antidepressant fluoxetine could restore CUMS-induced depression-like behavior in mice by significantly decreasing the level of NLRP3 and caspase 1 [77].

Cinnamic aldehyde reversed the loss of sucrose preference in CUMS rats and also reversed the increased COX-2 hippocampal expression and enzyme activity. However, no behavioral effects have been observed when mice injected with cinnamic aldehyde were subjected in the FST [78]. Restoration of PGE_2_ concentration in frontal cortex and hippocampus of stressed rats was also found in cinnamic aldehyde-treated animals [69]. It is interesting to mention that some of these compounds display notable anti-inflammatory actions, such as thymol [79] and geraniol [80], while others induce peripheral pro-inflammatory effects, e.g., cinnamic aldehyde [81]. Taken together, it is still unknown if a putative central nervous system anti-inflammatory effect is required for the antidepressant action of conventional drugs. However, further studies aimed to identify the molecular site of action of these compounds are mandatory.

Considering that most isolated constituents share a monoaminergic and/or pro-inflammatory mechanism of antidepressant action, it could be suggested that these compounds may present some chemical similarities, thus supporting an interaction with the same molecular site of action. However, the diversity of chemical structures of these compounds makes it difficult to establish a chemical template determining the antidepressant activity. This fact can be explained due to possible distinct molecular sites of action, or formation of psychoactive metabolite products.

### 6.2. Isolated Constituents without Antidepressant Mechanism of Action

Vanillin a constituent of essential oils used in cooking because of its pleasant odor and flavor to the food. This constituent reduced immobility duration in the FST and TST in mice after oral acutely administration. Chronic oral treatment with vanillin reduced immobility time in mice at significantly lower levels when compared to fluoxetine [75].

α-Asarone and β-asarone are found in several essential oils, including as major components from the rhizome essential oil of *Acorus tatarinowii* Schott. These isolated compounds as well the *Acorus tatarinowii* Schott essential oil displays antidepressant-like effect in the FST and TST [33]. No details about the antidepressant mechanisms of action of these isolated constituents were already proposed. Two other isolated constituents, limonene and α-phellandrene, reversed the depressant-like behavior and reduced nociceptive responses in rodents subjected to a model of neurophatic pain [46]. These effects are quite interesting and should be further investigated, since chronic pain is a common comorbidity of major depressive patients.

## 7. Conclusions

The antidepressant effects of essential oils and their constituents are very promising but they are still at the preliminary stages. Few clinical trials have been performed until now, and most of them are aimed for testing the effects of lavender oil. Importantly, the preclinical effects of lavender oil on rodents were superficially studied, and there is no suggestion of mechanism of action for this antidepressant effect. By contrast, some essential oil constituents display promising antidepressant effects by involving monoamine neurotransmission. With respect to the attribution of the antidepressant effect of a whole essential oil to one single constituent, the overall conclusion remains that the diversity of chemically active constituents of essential oils can be an advantage in the treatment of depression, since more than one compound with positive effects on depression can evoke synergic actions. Finally, the importance of these preclinical observations for the clinical overall picture of depression still needs to be addressed through further research. Future clinical trials will give scientific support for the employment of essential oils with potential antidepressant actions as real options for the treatment of depressive states.

## Figures and Tables

**Table 1:** Aromatic plant essential oils studied in experimental depression.

Essential Oils	Administration via and Duration of Treatment	Animal Specie	Dose Range Tested and Minimal Active Dose	Behavioral Test	Observed Effects	Mechanism of Action	Observations	Reference
*Acorus tatarinowii* Schott	Oral gavage, acute	ICR mouse	30–240 mg/kg (60 mg/kg)	FST, TST	Reduced immobility time in both assays		DR+	[33]
							U-inverted curve	
							Controls: negative and positive (imipramine)	
*Asarum heterotropoides* F. Schmidt	Inhalation, acute	ICR mouse	0.25–2.0 g (0.25 g)	FST, TST	Reduced immobility time in both tests	Reversed the increase of CRF- and TH-positive cells in the paraventricular nucleus, and locus coeruleus, respectively;	DR+ Controls: negative and positive (fluoxetine)	[34]
						Reversed the decrease of 5-HT-positive cells in the dorsal raphe nucleus		
*Citrus limon* (L.) Osbeck	Inhalation, acute	ICR mouse	Saturated chamber (90 min)	FST	Reduced immobility time	The treatment with flumazenil (GABA_A_ antagonist), buspirone (5-HT_1A_ partial agonist), DOI (5-HT_2A_ receptor agonist), miaserin (5-HT_2A/C_ receptor agonist), apomorphin (D receptor agonist) and haloperidol (D receptor antagonist) blocked the antidepressant effect. Increased hippocampal DA and prefrontal cortex and hippocampal 5-HT	DR−	[35]
							Controls: negative and positive (fluoxetine and imipramine)	
							Reduced spontaneous locomotor activity	
*Citrus limon* (L.) Osbeck	Inhalation, acute	SD rats	Saturated chamber (60 min)	FST	Reduced immobility time		DR−	[36]
							Controls: negative and positive (imipramine)	
							Reduced spontaneous locomotor activity	
*Citrus limon* (L.) Osbeck	Oral gavage, 30 days	Swiss mouse	50–150 mg/kg (50 mg/kg)	FST	Reduced immobility time		DR+	[37]
							Controls: negative and positive (imipramine and paroxetine)	
							The treatment decreased spontaneous locomotion increased sleeping duration	
*Eugenia uniflora* L.	Oral gavage, acute	Swiss mouse	1–50 mg/kg (10 mg/kg)	TST	Reduced immobility time	The blockade of 5-HT2_A/C_, α_1_ and α_2_-receptors prevented the antidepressant effects;	DR+ Controls: negative and positive (fluoxetine)	[38]
						In vitro inhibition of linoleic acid peroxidation;		
						Reduced SNP-induced lipoperoxidation in cortex, hippocampus and cerebellum		
*Lavandula angustifólia* Mill.	Intraperitoneal, acute	SD rat	5–20% (5%)	FST	Reduced immobility time		DR+	[39]
							Controls: negative and positive (fluoxetine and imipramine)	
*Lavandula angustifólia* Mill.	Inhalation, acute	ICR mouse	Saturated chamber (90 min)	FST	No effects were observed		DR−	[35]
							Controls: negative and positive (fluoxetine and imipramine)	
*Litsea glaucescens* Kunth	Intraperitoneal, three times within 24 h	ICR mouse	54.8–300 mg/kg (100 mg/kg)	FST	Reduced immobility time		DR+	[40]
							Controls: negative and positive (imipramine)	
*Mentha* × *piperita* L.	Inhalation, acute	ICR mouse (female)	Saturated chamber (10 min)	FST	Reduced immobility time		DR−	[41]
							Controls: negative	
*Perilla frutescens* L. Britton	Oral gavage, 3 weeks	ICR mouse	3–9 mg/kg (3 mg/kg)	CUMS, FST, TST, OFT	Restored sucrose preference in CUMS mice;	Reversed the 5-HT and 5-HIAA reduced concentrations in CUMS mice; Restored the serum IL-6, IL-1β, and TNF-α levels in CUMS mice	DR+	[42]
					Reverted the reduced spontaneous locomotion in CUMS mice;		U-inverted curve	
					Restored increased immobility time in CUMS mice		Controls: negative and positive (fluoxetine)	
*Perilla frutescens* L. Britton	Oral gavage, 3 and 4 weeks	ICR mouse	3–6 mg/kg (3 mg/kg)	CUMS, FST, sucrose preference	Restored the CUMS-induced decreased sucrose preference and increased immobility time	Restored the CUMS-induced reduction of hippocampal protein and mRNA BDNF	DR+	[43]
							Controls: negative and positive (fluoxetine)	
*Rosmarinus officinalis* L.	Oral gavage, acute	Swiss mouse	0.1–100 mg/kg (0.1 mg/kg)	TST	Reduced immobility time		DR+	[44,45]
							Controls: negative and positive (fluoxetine)	
*Rosmarinus officinalis* L.	Intraperitoneal, acute	SD rat	5–20% (5%)	FST	Reduced immobility time		DR+	[39]
							U-inverted curve	
							Controls: negative and positive (fluoxetine and imipramine)	
*Salvia sclarea* L.	Intraperitoneal and inhalation, acute	SD rat	5–20% (5%); satured chamber (1, 2, 4 and 6 h)	FST	Reduced immobility time when injected and inhaled	The pretreatment with haloperidol (Dopamine receptor antagonist), SCH-23390 (D1 receptor antagonist) and buspirone (5-HT_1A_ partial agonist) blocked the antidepressant effect	DR+	[39]
							Controls: negative and positive (fluoxetine and imipramine)	
*Schinus terebinthifolius* Raddi	Oral gavage, 15 days	Wistar rats	100 mg/kg	FST	Restored increased immobility time in rats subjected to a model of neuropathic pain		DR−	[46]
							Controls: negative and positive (ketamine)	
*Syzygium aromaticum* (L.) Merr, & L.M.Perry	Oral gavage, acute	ICR mouse	50–200 mg/kg (100 mg/kg)	FST, TST	Reduced immobility time in both tests		DR+	[47]
							Controls: negative and positive (imipramine) LD_50_ = 45564.556 g/kg (po)	
*Syzygium aromaticum* (L.) Merr, & L.M.Perry	Oral gavage, 5 weeks	SD rat	50–200 mg/kg (50 mg/kg)	CUMS, novelty-suppressed feeding behavior	Restored sucrose preference in CUMS rats; Reverted the increased latency to feed in a unfamiliar environment in CUMS rats	Restored hippocampal BDNF protein, p-ERK and p-CREB expression	DR+	[47]
							Controls: negative and positive (imipramine)	
*Thymus vulgaris* L. (Lamiaceae)	Inhalation, acute	ICR mouse (female)	Saturated chamber (10 min)	FST	Reduced immobility time		DR−	[41]
							Controls: negative	
*Toona ciliata* Roem. var. *yunnanensis* (C. DC.) C.Y. WU	Oral gavage, acute	ICR mouse	10–80 mg/kg (10 mg/kg)	FST, TST	Reduced immobility time in both tests		DR+	[48]
							Controls: negative and positive (imipramine)	
*Toona ciliata Roem* var. yunnanensis (C. DC.) C.Y. WU	Oral gavage, acute	SD rat	10–80 mg/kg (10 mg/kg)	CUMS	No behavioral effects were evaluated	Increased hippocampal monoamines (5-HT, NE and DA) and BDNF contents in CUMS rats;	DR+ Controls: negative and positive (imipramine)	[48]
						Reduced serum corticosterone in CUMS rats		
*Valeriana wallichii* DC.	Oral gavage, acute and 14 days	Albino Laca mouse (male and female)	10–40 mg/kg (10 mg/kg)	FST	Reduced immobility time	Increased noradrenaline and 5-HT levels after repeated administration; The acute antidepressant effect was prevented by pretreatment with L-arginine (NO precursor) and sildenafil (phosphodiesterase 5 inhibitor), while it was potentiated with L-NAME (NOS inhibitor) and methylene blue (inhibitor of soluble guanylate cyclase)	DR+	[49]
							Controls: negative and positive (imipramine)	
*Zingiber officinale* Roscoe	Inhalation, acute	ICR mouse (female)	Saturated chamber (10 min)	FST	Reduced immobility time		DR−	[41]
							Controls: negative	

FST: forced swimming test; TST: tail suspension test; OFT: open field test; CUMS: Chronic unpredictable mild stress; DR−: absence of dose/concentration response; DR+: dose/concentration response design; 5-HT: serotonin; DA: dopamine; NE: noradrenaline.

**Table 2:** Constituents from essential oils tested in experimental depression.

Constituents	Via of Administration and Duration of Treatment	Animal Specie	Dose Range Tested and Minimal Active Dose	Behavioral Test	Observed Effects	Mechanism of Action	Observations	Reference
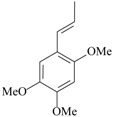	Intraperitoneal, acute	ICR mouse	5–20 mg/kg (10 mg/kg)	FST, TST	Reduced immobility time in both assays		DR+	[33]
Asarone							Controls: negative and positive (imipramine)	
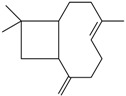	Intraperitoneal, acute	C57BL/6 mouse	50 mg/kg	FST, TST, novelty-suppressed feeding behavior	Reduced immobility time in the TST and the FST; decreased feeding latency in the novelty-suppressed feeding test	The pretreatment with AM630 (CB_2_ antagonist) prevented the anti-immobility effects	DR−	[52]
β-Caryophyllene							Controls: negative	
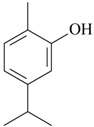	Oral gavage, acute	Swiss mouse	12.5–50 mg/kg (12.5 mg/kg)	FST, TST	Reduced immobility time in both tests	The pretreatment with SCH23390 (D_1_ antagonist) and sulpiride (D_2_ antagonist) prevented the anti-immobility effects	DR+	[68]
Carvacrol							Controls: negative and positive (imipramine)	
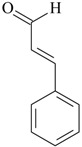	Oral gavage, 21 days	SD rat, 18 months old	22.5–90 mg/kg (45 mg/kg)	CUMS	Reversed decreased sucrose preference and spontaneous locomotion in CUMS rats	Reversed the increased hippocampal COX-2 protein and activity; Reversed the elevated PGE_2_ concentration in frontal cortex and hippocampus in CUMS rats	DR+	[69]
Cinnamic aldehyde							Controls: negative and positive (fluoxetine)	
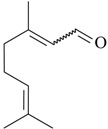	Inhalation, acute	SD rats	Saturated chamber (60 min)	FST	Reduced immobility time		DR−	[36]
							Controls: negative and positive (imipramine)	
Citral							Hypolocomotion	
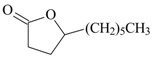	Intraperitoneal, acute	Wistar rat	0.1–0.3 g/kg	FST	No effects		DR+	[45,70]
γ-Decanolactone							Controls: negative Hypolocomotion at higher doses	
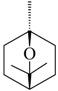	Intraperitoneal, three times within 24 h	ICR mouse	100 mg/kg	FST	No effects		DR−	[40]
Eucalyptol							Controls: negative and positive (imipramine)	
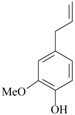	Intraperitoneal, 14 days	ddY mice	10–100 mg/kg (30 mg/kg)	FST, TST	Reduced immobility time in the TST and increased number of wheel rotations in the FST	Increased Hippocampal BDNF and metallothionein-III (brain-predominant protein that alleviates various neurotoxic events) mRNA	DR+	[61]
							Controls: negative and positive (imipramine)	
Eugenol	Oral, mixed with drinking water, 14 days	ICR mouse	0.17 mmol/kg	FST	Increased number of wheel rotations in the FST	Inhibits human MAO_A_ (IC50 34.4 µM) preferencially than MAO_B_ (IC_50_ 288 µM) activity	DR−	[62]
							Controls: negative	
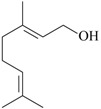	Oral gavage, 4 weeks	ICR mouse	20–40 mg/kg (20 mg/kg)	CUMS, FST, TST	Restored decreased sucrose preference and increased immobility time in the TST and FST in mice subjected to CUMS	Reversed the IL-1β-related CNS inflammation by markedly inhibiting CUMS-induced PFC NF-κB pathway and modulating NLRP3 inflammasome activation (activated caspase 1) in CUMS mice	DR+	[60]
Geraniol							Controls: negative and positive (fluoxetine)	
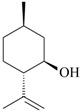	Intraperitoneal, acute	Swiss mouse	25–50 mg/kg (25 mg/kg)	FST, TST	Increased immobility time		DR+	[71]
Isopulegol							Controls: negative and positive (imipramine)	
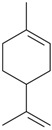	Oral gavage, 15 days	Wistar rat	10 mg/kg	FST	Restored increased immobility time in rats subjected to a model of neuropathic pain		DR−	[46]
							Controls: negative and positive (ketamine)	
Limonene	Intraperitoneal, three times within 24 h	ICR mouse	100 mg/kg	FST	No effects		DR−	[40]
							Controls: negative and positive (imipramine)	
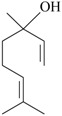 Linalool	Intraperitoneal, three times within 24 h	ICR mouse	54.8–173.2 mg/kg (100 mg/kg)	FST	Reduced immobility time		DR+	[40]
							U-inverted curve	
							Controls: negative and positive (imipramine)	
							The treatment reduced spontaneous locomotion	
	Intraperitoneal, three times within 24 h	ICR mouse	100 mg/kg	FST	Reduced immobility time	The pretreatment with WAY100,635 (5-HT_1A_ antagonist) and yohimbine (α_2_-antagonist) prevented the antidepressant-like effects	DR−	[58]
							Controls: negative and positive (imipramine)	
	Intraperitoneal, acute	Swiss mouse	10–200 mg/kg (100 mg/kg)	TST	Reduced immobility time		DR+	[59]
							Controls: negative and positive (imipramine)	
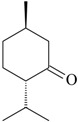	Oral gavage, 3 weeks	ICR mouse	15–30 mg/kg (15 mg/kg)	CUMS, FST, TST	Reversed the decrease of sucrose consumption, the hypolocomotion and the increased immobile time in the TST and FST in CUMS mice	Restored the CUMS-induced reductions in hippocampal NE and 5-HT levels; Reverted the increased hippocampal pro-inflammatory cytokines levels (IL-1β, IL-6, and TNFα) in CUMS mice; Inhibited the increased hippocampal nod-like receptor protein 3 (NLRP3) inflammasome, and caspase-1 protein expression in CUMS mice	DR+	[72]
Menthone							Controls: negative and positive (fluoxetine)	
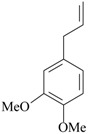	Oral gavage, acute	Wistar rats	1.0–10.0 µl/mL/kg (1.0 µl/mL/kg)	FST	Reduced immobility time		DR+	[50]
Methyl-eugenol							Controls: negative	
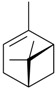	Intraperitoneal, three times within 24 h	ICR mouse	100 mg/kg	FST	No effects		DR−	[40]
α-Pinene							Controls: negative and positive (imipramine)	
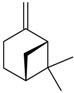 β-Pinene	Intraperitoneal, three times within 24 h	ICR mouse	54.8–173.2 mg/kg (100 mg/kg)	FST	Reduced immobility time		DR+	[40]
							Controls: negative and positive (imipramine)	
							The treatment reduced the spontaneous locomotion	
	Intraperitoneal, three times within 24 h	ICR mouse	100 mg/kg	FST	Reduced immobility time	The pretreatment with WAY100,635 (5-HT_1A_ antagonist), propranolol (β-antagonist), DSP-4 (NE neurotoxin), SCH23390 (D_1_ antagonist) prevented the anti-immobility effect	DR−	[58]
							Controls: negative and positive (imipramine)	
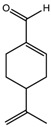	Oral gavage, 7 days	ICR mouse	60–120 mg/kg (60 mg/kg)	LPS-induced depressant-like behavior, FST and TST	Reversed increased in immobility time in the FST and TST in LPS-treated mice	Reversed the reduced concentrations of 5-HT and NE, and attenuated LPS-induced increases of serum protein levels and prefrontal cortex mRNA of TNF-α and IL-6	DR+	[70]
							Controls: negative and positive (fluoxetine)	
Perillaldehyde	Inhalation, 9 days	ddY mouse	0.1–10% dropped on the area between eyes and nose (1%)	CUMS, FST	Reduced immobility time in naïve mouse and reversed increased immobility time in CUMS mice		DR+	[57]
							Controls: negative and positive (minalcipran)	
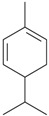	Oral gavage, once daily, 15 days	Wistar rat	10 mg/kg	FST	Restored increased immobility time in rats subjected to a model of neuropathic pain		DR−	[46]
α-Phellandrene							Controls: negative and positive (ketamine)	
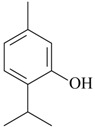	Oral gavage, 3 weeks	ICR mouse	15–30 mg/kg (15 mg/kg)	CUMS, TST, FST	Reversed the decrease of sucrose consumption, the loss of body weight, and the increased immobile time in the TST and FST in CUMS mice	Restored the CUMS-induced reductions in hippocampal NE and 5-HT; Reverted the increased hippocampal mRNA of pro-inflammatory cytokines (IL-1β, IL-6, and TNFα) in CUMS mice; Inhibited the activation of nod-like receptor protein 3 (NLRP3) inflammasome and its adaptor, and subsequently decreased the expression of caspase-1	DR+	[73]
Thymol							Controls: negative and positive (fluoxetine)	
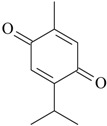	Intraperitoneal, acute	Swiss mouse	20 mg/kg	FST, TST	Reduced immobility time in both tests	A significant elevation of 5-HT whole brain levels was observed; Increased glutathione levels and decreased TBARS levels in the whole brain	DR−	[74]
Thymoquinone							Controls: negative and positive (fluoxetine)	
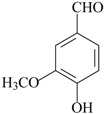	Oral gavage, acute and 10 days	Swiss mouse (male and female)	10–100 mg/kg (10 mg/kg)	FST, TST	Reduced immobility time under acute and chronic treatments		DR+	[75]
Vanillin							Controls: negative and positive (fluoxetine and imipramine)	

FST: forced swimming test; TST: tail suspension test; OFT: open field test; CUMS: Chronic unpredictable mild stress; DR+: dose/concentration response design; DR−-: absence of dose/concentration response; 5-HT: serotonin; DA: dopamine; NE: noradrenaline.
